# Evaluation of Hemodynamics in Focal Steatosis and Focal Spared Lesion of the Liver Using Contrast-Enhanced Ultrasonography with Sonazoid

**DOI:** 10.1155/2014/604594

**Published:** 2014-08-04

**Authors:** Kazue Shiozawa, Manabu Watanabe, Takashi Ikehara, Michio Kogame, Mie Shinohara, Masao Shinohara, Koji Ishii, Yoshinori Igarashi, Hiroyuki Makino, Yasukiyo Sumino

**Affiliations:** ^1^Division of Gastroenterology and Hepatology, Department of Internal Medicine, Toho University Medical Center, Omori Hospital, 6-11-1 Omorinishi, Ota-ku, Tokyo 143-8541, Japan; ^2^Division of Gastroenterology and Hepatology, Department of Internal Medicine, Saiseikai Yokohamashi Tobu Hospital, 3-6-1 Shimosueyoshi, Tsurumi-ku, Yokohama-shi, Kanagawa 230-0012, Japan

## Abstract

We aim to investigate the hemodynamics in focal steatosis and focal spared lesion of the liver using contrast-enhanced ultrasonography (CEUS) with Sonazoid. The subjects were 47 patients with focal steatosis and focal spared lesion. We evaluated enhancement patterns (hyperenhancement, isoenhancement, and hypoenhancement) in the vascular phase and the presence or absence of a hypoechoic area in the postvascular phase for these lesions using CEUS. Of the 24 patients with focal steatosis, the enhancement pattern was isoenhancement in 19 and hypoenhancement in 5. Hypoechoic areas were noted in the postvascular phase in 3 patients. Of the 23 patients with focal spared lesions, the enhancement pattern was isoenhancement in 18 and hyperenhancement in 5. No hypoechoic areas were noted in the postvascular phase in any patient. The hemodynamics in focal steatosis and focal spared lesions in nondiffuse fatty liver can be observed using low-invasive procedures in real-time by CEUS. It was suggested that differences in the dynamics of enhancement in the vascular phase of CEUS were influenced by the fat deposits in the target lesion, the surrounding liver parenchyma, and the third inflow.

## 1. Introduction

Nondiffuse fatty liver is considered to develop when the fat accumulation ability of hepatocytes or fat deposition becomes heterogeneous throughout the liver and is classified into the following 4 types: focal steatosis, multifocal steatosis, lobar or segmental steatosis, and focal spared lesion in fatty liver [1]. Based on imaging studies, insulin and nutrient levels in the blood flowing into the corresponding hepatic areas are considered to be causative in the frequent development of focal steatosis and focal spared lesion [2].

Some cases of focal steatosis and focal spared lesion exhibit an oval or mass-like appearance on imaging, and differentiation from liver tumors may be problematic. In this study, we performed contrast-enhanced ultrasonography (CEUS) for focal steatosis and focal spared lesions using Sonazoid (Daiichi Sankyo Pharmaceutical, Tokyo, Japan), in order to diagnose liver mass lesions, and obtained information on the hemodynamics of focal steatosis and focal spared lesions. Herein, we report these data with a review of the literature.

## 2. Materials and Methods

The subjects were 47 patients in whom liver mass lesions were detected on abdominal ultrasonography during health check-up and underwent CEUS with Sonazoid. The findings of these patients were investigated retrospectively. These were diagnosed with focal steatosis (24 cases) or a focal spared lesion (23 cases) on dynamic computed tomography (CT) and magnetic resonance imaging [3, 4]. At first, the presence or absence of an existing blood vessel within the lesion was evaluated by color doppler ultrasonography (CDUS). Next, CEUS was performed using an Aplio XG (Toshiba Medical Systems, Tokyo, Japan) with a convex probe (PVT-375BT, 3.75 MHz center frequency). The mechanical index (MI) for the acoustic output was set to 0.2, and a single focus point was set at the lower margin of the lesion. An intravenous bolus injection of Sonazoid (0.5 mL) was administered via a left cubital venous line followed by flushing with 10 mL of normal saline. The lesion was evaluated with regard to the following findings: the dynamics of the enhancement in the vascular phase (0–40 seconds), the presence or absence of an existing blood vessel within the lesion in the vascular phase, and the presence or absence of a hypoechoic area in the postvascular phase (after 15 minutes). Digital cine clips of the CEUS images were stored on the hard disk of the scanner and transferred to a high performance personal computer for subsequent analysis. The dynamics of the enhancement in the vascular phase were compared with those in the surrounding liver and classified into the following 3 enhancement patterns: hyperenhancement, isoenhancement and hypoenhancement. The postvascular phase was observed using the advanced dynamic flow (ADF) mode in some cases. Using the ADF mode, Sonazoid bubbles phagocytosed by Kupffer cells were destroyed, with a high MI of 1.0 [5]. Since the presence or absence of hypoechoic areas in the postvascular phase cannot be readily identified, particularly in high-echoic lesions, this procedure is useful for such determination.

All CEUS images were reviewed by two hepatologists, one was with 15 years of experience and other was with 27 years of experience.

### 2.1. Statistical Analysis

The presence or absence of an existing blood vessel in the lesion and the enhancement pattern (hyperenhancement, isoenhancement, and hypoenhancement) in the vascular phase were compared using Fisher's exact test. Statistical analyses were performed using SPSS version 11.0 (Statistical Package for the Social Sciences) for Windows (Microsoft) and *P* < 0.05 was considered to indicate a statistically significant difference.

This study was approved by the Ethical Review Board of Toho University Medical Center, Omori Hospital.

## 3. Results

### 3.1. Focal Steatosis

Of the 24 patients with focal steatosis (Table 1), 13 and 11 were male and female, respectively, and the mean age was 61.8 ± 14.3 years (median: 64.5 years). The mean major axis of the lesion was 20.0 ± 9.5 mm (median: 19.5 mm) and the location was S3 in 1 patient (near the umbilical portion), S4 in 16 (transverse part of the portal vein in 8, near the falciform ligament of the liver (Sappey's vein area) in 6, and the gallbladder bed and adjacent to IVC in 1 each), S5 in 6 (the gallbladder bed in 3, the right lobe surface in 1, and other area of the right lobe in 2), and S8 (right lobe surface) in 1.

**Figure 1:**
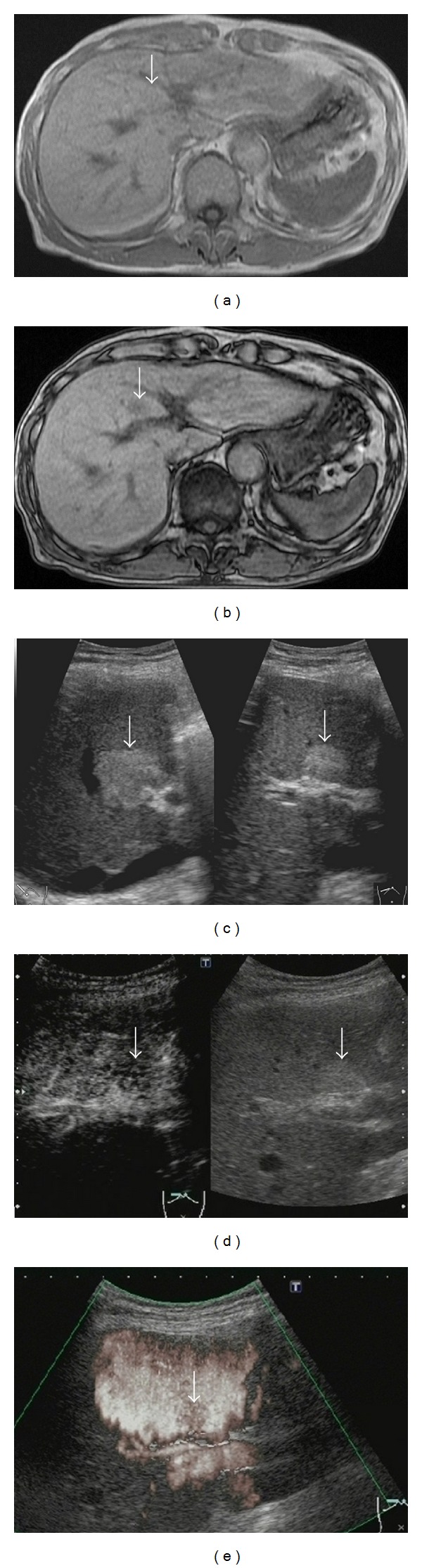
A 75-year-old male (case no. 24) with focal steatosis in the S4 (transverse portion). (a) In-phase T1-weighted magnetic resonance image (MRI) scan shows the isointense lesion (arrow) in the S4 (transverse portion). (b) The lesion is hypointense on an out of-phase T1-weighted MRI scan (arrow) and is diagnosed as focal steatosis. (c) Gray-scale ultrasonography (US) (right intercostal axis) shows a hyperechoic lesion (arrow). (d) Left: in the vascular phase (17 seconds) of contrast-enhanced ultrasonography (CEUS) (right subcostal axis), the lesion (arrow) is hypoenhancement compared with the surrounding liver. Right: gray-scale US (monitor mode) (arrow). (e) In the advanced dynamic flow (ADF) mode of the postvascular phase of CEUS (right intercostal axis), the lesion shows a hypoechoic area (arrow).

The enhancement pattern in the vascular phase of CEUS was isoenhancement in 19 patients and hypoenhancement in 5 (Figure 1). An existing blood vessel was noted in the lesion in the vascular phase in 7 patients. On comparison of the enhancement patterns, the patterns were isoenhancement and hypoenhancement of the lesions in 5 and 2 of the 7 patients with existing blood vessels, respectively, and in 14 and 3 of the lesions of the 17 patients with no existing blood vessels, respectively, showing no significant difference.

A hypoechoic area was noted in the postvascular phase in 3 patients, which was also evident using the ADF mode in these same 3 patients.

### 3.2. Focal Spared Lesion

Of the 23 patients with focal spared lesions (Table 2), 15 and 8 were male and female, respectively, and the mean age was 52.9 ± 14.1 years (median: 52 years). The mean major axis of the lesion was 22.8 ± 11.8 mm (median: 20 mm), and the location was S3 in 1 patient (near the umbilical portion), S4 in 5 (transverse part of the portal vein in 3, and Sappey's vein area and the gallbladder bed in 1 each), S5 in 7 (the gallbladder bed in 6, and the right lobe surface in 1), S6 in 5 (the right lobe surface in 4, and the right hepatic hilum in 1), S7 (right lobe surface) in 2, and S8 in 3 (the right lobe surface in 1, and other area of the right lobe in 2).

**Figure 2:**
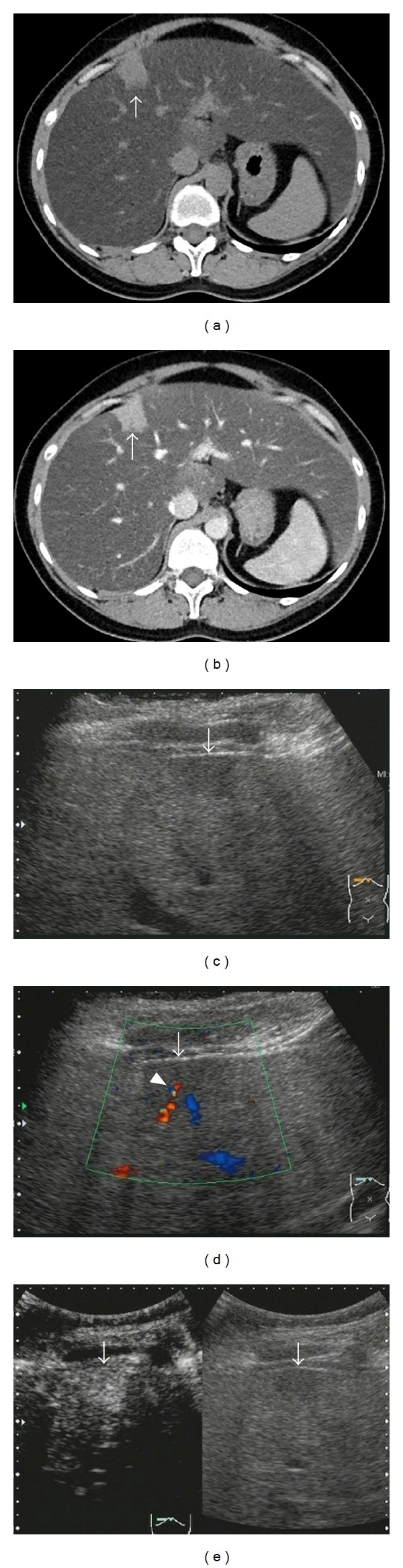
A 35-year-old female (case No. 5) with focal spared lesion in the S4 (near the falciform ligament of liver so called Sappey's vein area). (a) Unenhanced computed tomography (CT) scan shows a hyperattenuating lesion (arrow) in the S4. (b) Enhanced CT scan shows the lesion (arrow) of hyperattenuation in the portal phase. (c) Gray-scale US (right intercostal axis) shows a hypoechoic lesion (arrow). (d) Color Doppler ultrasonography (CDUS) (right intercostal axis) shows hepato petal flow (arrow head) toward the lesion (arrow). (e) Left: In the vascular phase (13 seconds) of CEUS (right intercostal axis), the lesion (arrow) is hyperenhancement compared with the surrounding liver. Right: Gray-scale US (monitor mode) (arrow).

The enhancement pattern in the vascular phase of CEUS was isoenhancement of the lesion in 18 patients and hyperenhancement of the lesion in 5 (Figure 2).

An existing blood vessel was noted in the lesion in the vascular phase in 5 patients. On comparison of the enhancement patterns, the patterns were hyperenhancement and isoenhancement of the lesions in 3 and 2 of the 5 patients with existing blood vessels, respectively, and in 2 and 16 of the lesions of the 18 patients with no existing blood vessels, respectively, showing a significant difference (*P* = 0.048), and existing blood vessels were more often noted in the cases with hyperenhancement.

No hypoechoic areas were noted in the postvascular phase in any patient. Nine patients were observed using the ADF mode, but no hypoechoic areas were noted.

## 4. Discussion

Fatty liver is caused by the accumulation of triglycerides and other nutrients within hepatocytes and is roughly divided into diffuse and nondiffuse fatty liver. Nondiffuse fatty liver is further classified into 4 types: focal steatosis, multifocal steatosis, lobar or segmental steatosis, and focal spared lesion in the fatty liver [1]. The characteristic hemodynamic profile of the liver is considered to be involved in the maldistribution of fat in the liver parenchyma. The blood supply to the liver is derived from the hepatic artery and portal vein. The portal vein supplies 70% to 80% of the blood, delivering nutrients and other substances derived from the intestine [6]. It has been reported that, in nondiffuse fatty liver, localized fat and nonfat deposition are likely to occur in specific lesions, such as the dorsal S4 lesion of the liver, around the gallbladder and Sappey's vein area [7–9]. Matsui et al. [2, 10] investigated many cases using CT during arterial portography (CTAP) and clarified that the above-described lesions, in which fat deposition/nonfat deposition are likely to occur, exhibit defects of enhancement on CTAP, in the absence of any apparent lesion, that is, the defects correspond to pseudolesions, and the presence of a blood vessel (third inflow) flowing into the liver other than the portal vein is involved in the appearance of the pseudolesions. The third inflow includes the right gastric vein, cystic vein, and Sappey's vein [11]. Since the portal blood flow is important in the transport of dietary fat from the intestine, the concentrations of hormones, such as insulin, and nutrients in the third inflow, could be different from those in the portal blood, and these differences could contribute to the heterogeneous deposition of fat [2].

For focal steatosis and focal spared lesions, hemodynamics has been reported on CEUS using SonoVue (Bracco, Milan, Italy) [12–14]. However, to our knowledge, there has been no report on the use of CEUS using Sonazoid. CEUS using SonoVue can only be used to evaluate the vascular phase. In contrast, the vascular and postvascular phase (Kupffer phase) can be evaluated with CEUS using Sonazoid based upon the characteristic of Sonazoid following phagocytosis by Kupffer cells.

Liu et al. [12] reported the hemodynamics of 25 lesions in 20 patients with focal steatosis by CEUS using SonoVue, in which hypoenhancement and isoenhancement of the lesions were observed in the vascular phase in 44% each and hyperenhancement was observed in 12%. In our study, isoenhancement and hypoenhancement of the lesions were noted in 19 (79%) and 5 (21%) of the 24 patients, and no hyperenhancement was observed in any patient. Considering the findings of Liu et al., it was suggested that focal steatosis tends to present as hypoenhanced or isoenhanced lesions in the vascular phase of CEUS.

Liu et al. also investigated the time required for contrast agent to reach and enter the lesion with focal steatosis [12]. The time to reach the hypoenhanced lesions, which was 1–21 seconds, was delayed compared with the time (1–7 seconds) taken for the surrounding liver parenchyma. They did not mention the reason for this, but, in all likelihood, the staining intensity or speed may have tended to decrease in the hypoenhanced regions compared to that in the surrounding liver parenchyma due to the exclusion/narrowing of blood vessels by the fatty cells; that is, exclusion of the sinusoid by fat droplets [15]. Therefore, hyperenhancement is unlikely to occur in focal steatosis, and the cause of hyperenhancement in the 12% in the study reported by Liu et al. remains unclear.

Among the 23 patients with focal spared lesions, isoenhancement and hyperenhancement of the lesions were noted in the vascular phase of CEUS in 18 and 5 patients, respectively, and no hypoenhancement was noted in any patient. In the focal steatosis cases described above, enhancement tended to decrease in the focal steatosis lesion, compared with that in the surrounding liver parenchyma, due to the influence of fatty cells. In these focal spared lesions, isoenhancement was noted in most cases, but hyperenhancement was noted in some cases. This may have been due to fatty cell-induced exclusion and narrowing of blood vessels in the surrounding liver parenchyma by fatty cells [16, 17], in contrast to focal steatosis, and enhancement of the lesion may have appeared to be increased relative to that of the surrounding liver parenchyma. In normal liver parenchyma, arterial blood generally flows in first, followed by perfusion via the portal blood flow. Since ectopic venous circulation other than the portal vein, that is, the third inflow, is involved in focal spared lesions, the third inflow may have entered the focal spared lesion earlier than the portal blood perfusion within the liver parenchyma, resulting in more rapid and stronger enhancement compared with the surrounding liver parenchyma.

Regarding the existing blood vessels and the enhancement pattern in focal steatosis and focal spared lesions, no significant correlation was noted between the presence of an existing blood vessel and the enhancement pattern in focal steatosis. In contrast, in focal spared lesions, existing blood vessels were noted in 3 of the 5 cases with hyperenhancement, showing a significant difference. The presence or absence of an existing blood vessel may influence the enhancement dynamics in focal spared lesions.

In the postvascular phase, hypoechoic areas were noted in 3 of the 24 focal steatosis cases, and these hypoechoic areas were also observed using the ADF mode in each of these cases. In contrast, no hypoechoic areas were noted in any of the 23 cases of focal spared lesion or of the 9 cases observed using the ADF mode. These findings suggest that the phagocytic ability of Kupffer cells is reduced in fat-containing cells compared with normal liver cells.

It was clarified that hemodynamics in lesions can be observed using low-invasive procedures in real-time by CEUS using Sonazoid in comparison with the previous CT angiography. And it was suggested that Kupffer function (phagocytosis) in the lesions could be evaluated by observing the postvascular phase. In addition, hypoechoic areas were noted in some of the focal steatosis cases in the postvascular phase. We require attention for differentiation of these lesions and malignant tumors.

Among the limitations, this study was retrospective in nature, that is, the number of cases was small, the lesions were not diagnosed histologically, and it was unclear whether the existing blood vessel within the lesions represented an artery, vein, or portal vein. It may be necessary to increase the number of cases and perform further evaluations including histopathological investigation.

## 5. Conclusion

We investigated the hemodynamics in focal steatosis and focal spared lesions in nondiffuse fatty liver by CEUS using Sonazoid. It was suggested that differences in the dynamics of enhancement in the vascular phase of CEUS were influenced by fat deposits in the target lesion and the surrounding liver parenchyma and the third inflow and that the fat deposition-associated phagocytic ability of Kupffer cells is involved with the presence or absence of the hypoechoic area in the postvascular phase.

As for the knowledge provided in this study, it will seem that it is necessary in future in distinguishing a malignant tumor from these lesion.

## Figures and Tables

**Table 1:** Of the 24 lesions with focal steatosis, patients character, location, size, enhancement pattern in the vascular phase of CEUS, presence of existing blood vessel in the CDUS and/or the vascular phase, presence of hypoechoic area in the postvascular phase, and ADF mode of CEUS.

No.	Age	Sex	Location	Size (mm)	Vessels seen on CDUS or/and CEUS	CEUS	
						Vascular	Postvascular/ADF
1	60	F	S4/Transverse portion	28	+	Iso	−∗
2	73	F	S4/Sappey's vein area	25	+	Hypo	−
3	72	M	S5/GB bed	25	+	Iso	−
4	80	M	S4/Transverse portion	36	+	Iso	−
5	45	M	S4/GB bed	8	+	Iso	+
6	49	M	S4/Sappey's vein area	21	+	Iso	−
7	62	M	S4/Sappey's vein area	15	+	Hypo	−
8	76	F	S4/Transverse portion	20		Iso	−
9	85	F	S3/Umbilical portion	7		Iso	−
10	59	M	S4/Transverse portion	18		Hypo	−
11	48	F	S4/Sappey's vein area	12		Iso	−
12	68	F	S4/Transverse portion	14		Iso	−
13	67	F	S4/Adjacent to IVC	40		Iso	−
14	50	M	S5/Right lobe surface	15		Iso	−
15	31	M	S8/Right lobe surface	8		Iso	−
16	66	F	S5/GB bed	30		Iso	−
17	74	M	S4/Transverse portion	8		Iso	−
18	80	F	S4/Transverse portion	27		Iso	−
19	64	M	S4/Sappey's vein area	31		Iso	−
20	45	F	S4/Sappey's vein area	20		Iso	−
21	65	M	S5/GB bed	19		Hypo	−
22	40	M	S5/Other area of the right lobe	15		Iso	−
23	49	F	S5/Other area of the right lobe	8		Iso	+
24	75	M	S4/Transverse portion	30		Hypo	+

GB: gall bladder, Iso: isoenhancement, hypo: hypoenhancement, ADF: advanced dynamic flow.

∗−: no hypoechoic area, +: hypoechoic area.

**Table 2:** Of the 23 lesions with focal spared lesions, patients character, location, size, enhancement pattern in the vascular phase of CEUS, presence of existing blood vessel in the CDUS and/or the vascular phase, presence of hypoechoic area in the postvascular phase, and ADF mode of CEUS.

No.	Age	Sex	Location	Size (mm)	Vessels seen on CDUS or/and CEUS	CEUS	
						Vascular	Postvascular/ADF
1	44	M	S4/GB bed	60.9	+	hyper	−∗
2	75	F	S5/GB bed	12	+	Iso	−
3	69	F	S6/Right hepatic hilum	14	+	hyper	−
4	48	M	S5/GB bed	12	+	Iso	−
5	35	F	S4/Sappey's vein area	21	+	hyper	−
6	52	M	S5/GB bed	17		Iso	−
7	45	M	S6/Right lobe surface	19		Iso	−
8	70	M	S7/Right lobe surface	41		Iso	−
9	37	F	S6/Right lobe surface	20		Iso	−
10	62	F	S8/Other area of the right lobe	18		Iso	−
11	52	M	S5/Right lobe surface	12		hyper	−
12	59	M	S8/Right lobe surface	23		hyper	−
13	64	M	S4/Transverse portion	22		Iso	−
14	45	M	S5/GB bed	18		Iso	−
15	24	M	S5/GB bed	20		Iso	−
16	71	F	S8/Other area of the right lobe	15		Iso	−
17	47	F	S3/Umbilical portion	13		Iso	−
18	59	M	S4/Transverse portion	40		Iso	−
19	34	M	S4/Transverse portion	20		Iso	−
20	36	M	S6/Right lobe surface	30		Iso	−
21	60	M	S7/Right lobe surface	35		Iso	−
22	56	M	S6/Right lobe surface	27		Iso	−
23	72	F	S5/GB bed	14		Iso	−

GB: gall bladder, hyper: hyperenhancement, Iso: isoenhancement, ADF: advanced dynamic flow.

∗−: no hypoechoic area.

## Abbreviations

CEUS: Contrast-enhanced ultrasonography
CT: Computed tomography
CDUS: Color Doppler ultrasonography
MI: Mechanical index
ADF: Advanced Dynamic Flow
CTAP: CT during arterial portography.
